# Supplementary material for: Relationships between Sleep Quality, Introspective Accuracy, and Confidence Differ among People with Schizophrenia, Schizoaffective Disorder, and Bipolar Disorder with Psychotic Features
Source: Behav Sci (Basel). 2024 Feb 28;14(3):192. doi: 10.3390/bs14030192 (PMC10968649; doi:10.3390/bs14030192)
Supplement: Supplementary file 1 [file behavsci-14-00192-s001.zip › behavsci-2879382-supplementary.pdf]

Supplemental Table S1

Average sleep quality scores and group comparisons between Non-SI and SI groups

	**Non-SI** (n = 95)	**SI** (n = 83)	
	M (SD)	*M (SD)*	*t*
PSQI Total	8.83 (4.23)	10.04 (3.96)	-1.95, p =0.05
PSQI Component 1	1.11 (0.92)	1.52 (0.94)	-2.96, p =0.004
PSQI Component 2	1.74 (1.07)	1.64 (1.04)	0.62, p = 0.54
PSQI Component 3	0.78 (0.99)	1.10 (1.19)	-1.92, p = 0.06
PSQI Component 4	1.09 (1.12)	0.85 (1.11)	1.37, p = 0.17
PSQI Component 5	1.42 (0.66)	1.66 (0.75)	-2.28, p = 0.02
PSQI Component 6	1.29 (1.37)	1.39 (1.37)	-0.44, p = 0.66
PSQI Component 7	1.25 (1.00)	1.40 (0.90)	-1.01, p = 0.31

Note. Non-SI = non-suicidal group; SI = actively suicidal group; PSQI Total = Global PSQI Score; PSQI Component 1 = Subjective sleep quality; PSQI Component 2 = Sleep latency; PSQI Component 3 = Sleep duration; PSQI Component 4 = Sleep efficiency; PSQI Component 5 = Sleep disturbances; PSQI Component 6 = Use of sleeping medication; PSQI Component 7 = Daytime dysfunction
